# Reliability and validity of the Brief Attention and Mood Scale of 7 Items: a self-administered, online assessment

**DOI:** 10.3389/fpsyg.2025.1579235

**Published:** 2025-10-01

**Authors:** Kevin P. Madore, Allen M. Osman, Kelsey R. Kerlan, Robert J. Schafer

**Affiliations:** Department of Research and Development, Lumos Labs, Inc., San Francisco, CA, United States

**Keywords:** brief attention and mood scale of 7 items, BAMS-7, validity, reliability, ROC, brain training, cognition, emotion

## Abstract

Changes in technology, regulatory guidance, and COVID-19 have spurred an explosion in online studies in the social and clinical sciences. This surge has led to a need for brief and accessible instruments that are designed and validated specifically for self-administered, online use. Addressing this opportunity, the Brief Attention and Mood Scale of 7 Items (BAMS-7) was developed and validated in six cohorts across four studies to assess real-world attention and mood in one instrument. In Study 1, an exploratory factor analysis was run on responses from an initial nine-item survey in a very large, healthy, adult sample (*N* = 75,019, ages 18–89 years). Two brief subscales comprising seven items total were defined and further characterized: one for Attention, the other for Mood. Study 2 established convergent validity with existing questionnaires in a separate sample (*N* = 150). Study 3 demonstrated known-groups validity of each subscale using a large sample (*N* = 58,411) of participants reporting a lifetime diagnosis of ADHD, anxiety, or depression, alongside the healthy sample of Study 1. The Attention subscale had superior discriminability for ADHD and the Mood subscale for anxiety and depression. Study 4 applied confirmatory factor analysis to data (*N* = 3,489) from a previously published cognitive training study that used the initial nine-item survey, finding that the Attention and Mood subscales were sensitive to the intervention (compared to an active control) to different degrees. In sum, the psychometric properties and extensive normative data set (*N* = 75,019 healthy adults) of the BAMS-7 may make it a useful instrument in assessing real-world attention and mood.

## 1 Introduction

Cognition and mood are impacted by numerous medical conditions (Armstrong and Okun, 2020; Bar, 2009; Eyre et al., 2015; Fast et al., 2023), lifestyle choices (Santos et al., 2014; Sarris et al., 2020; van Gool et al., 2007), healthy development and aging (Fernandes and Wang, 2018; Mather and Carstensen, 2005; Tomaszewski Farias et al., 2024; Yurgelun-Todd, 2007), and medications or other interventions (Keshavan et al., 2014; Koster et al., 2017; Reynolds et al., 2021; Skirrow et al., 2009). Conditions principally defined by impaired cognition – such as ADHD or mild cognitive impairment – are often associated with concomitant changes in mood status, either directly or indirectly (Chen et al., 2018; D’Agati et al., 2019; Ismail et al., 2017; Retz et al., 2012; Schnyer et al., 2015; Robison et al., 2020; Yates et al., 2013). Similarly, conditions principally defined by one’s mood or emotions – such as anxiety or depression – often have a corresponding impact on cognition (Gkintoni and Ortiz, 2023; Gulpers et al., 2016; Keller et al., 2019; Williams, 2016). The prevalence of cognitive-affective interactions underscores the dynamic coexistence between the two and has motivated the development of theoretical frameworks [e.g., Bar (2009)] and hypothesized mechanisms [e.g., Keller et al. (2019)] explaining their interdependence. Given the intimate relationship between cognition and mood, the ability to measure both in one scale may yield a more complete picture of functioning in clinical and psychological research.

Intersecting with the need for concurrent measurement of cognition and mood is a shifting research landscape. Due to advances in technological capabilities, changes in emphases in regulatory guidance, and lasting impacts of the COVID-19 pandemic, there has been a recent surge of online studies in the social and clinical sciences (Arechar and Rand, 2021; Goodman and Wright, 2022; Lourenco and Tasimi, 2020; Saragih et al., 2022). Related to this point is a growing body of contemporary research indicating that the psychometric properties of survey instruments may indeed be impacted by factors such as mode of administration and length (Aiyegbusi et al., 2024; Aiyegbusi et al., 2022; Gottfried, 2024; Kelfve et al., 2020; Lantos et al., 2023; Zager Kocjan et al., 2023; Wilson et al., 2024). As a result, instruments that are designed and characterized specifically for online research are paramount. To collect reliable responses from large numbers of participants via their own internet-connected devices, instrument qualities like brevity and accessibility of language are likewise required. For studies using relatively brief interventions, the time interval for evaluation is also important: for example, using Broadbent’s Cognitive Failures Questionnaire (CFQ) [e.g., Bridger et al. (2013), Broadbent et al. (1982), Goodhew and Edwards (2024), and Rast et al. (2009)] to evaluate cognitive failures over the past six months may not be appropriate for measuring change over a shorter period of time. Furthermore, instruments that have been normed and validated based on traditional, in-person administration may have different characteristics with at-home, self-administration on one’s own computer or smart device.

To meet the needs of the present-day landscape, and to offer a very large normative data set to the research community, we present and describe a brief, seven-item scale of real-world attention and mood: the BAMS-7. Unlike existing instruments that were developed for in-person administration or validated with relatively small samples, the BAMS-7 was designed from the outset for self-administration via personal digital devices and characterized using one of the largest normative datasets available (*N* = 75,019 evaluable participants). This combination of format, scalability, and psychometric validation addresses limitations in current tools and reflects growing trends in decentralized, online psychological research. The BAMS-7 complements existing instruments in the literature by emphasizing brevity, accessibility, and measures of multiple constructs (attention and mood) within one scale in a validated online format. Given that attention and mood are correlated in healthy (Carriere et al., 2008; Hobbiss et al., 2019; Irrmischer et al., 2018) and clinical populations (Bar, 2009; Skirrow et al., 2009; D’Agati et al., 2019; Retz et al., 2012), it may be advantageous to adopt one scale with separable measures of attention and mood. The scale may be useful both as an outcome measure (i.e., a dependent variable) or covariate (i.e., an independent variable) in clinical and psychological research.

In 2015, Hardy et al. (2015) published the results of a large, online study evaluating an at-home, computerized cognitive training program [described also in Ng et al. (2021)]. As a secondary outcome measure, the authors created a nine-item survey of “cognitive failures and successes as well as emotional status” [p. 6; 45]. This original survey is shown in Table 1 and consisted of two parts. In a first section of four items, participants responded to questions about the frequency of real-world cognitive failures or successes within the last month. In a second section of five items, participants responded to questions about the extent of agreement with statements relating to feelings of positive or negative mood and emotions, creativity, and concentration within the last week. Responses were on a five-point Likert scale and translated to score values of 0 to 4. Hardy et al. (2015) created a composite measure – the “aggregate rating” – by averaging across items.

**Table 1:** Original (Hardy et al., 2015) nine-item survey.

Item	During the past month, how often have you… *Valid response options: “Never,” “1–2 times during the month,” “1–2 times per week,” “Several times per week,” “Almost every day”*	Reverse-coded
1	…lost track of details as you were reading and needed to go back and reread sections?	X
2	…misplaced items (e.g., reading glasses, keys) around the house?	X
3	…found yourself losing concentration during a conversation?	X
4	…remembered someone’s name who had just been introduced to you?	

	Rate your experience over the last week… *Valid response options: “Strongly disagree,” “Disagree,” “Neither agree nor disagree,” “Agree,” “Strongly agree”*	
5	I felt creative.	
6	My ability to concentrate was good.	
7	I felt anxious.	X
8	I was in a bad mood.	X
9	I felt sad for no obvious reason.	X

Although the survey was not formally characterized, the first four items were similar to ones from the CFQ, and all items had a degree of face validity. Key differences from CFQ items reflected updates for modern-day relevance (e.g., removing “newspaper” as an example item that might be misplaced around the home) and for shortening the time interval of interest to make it possible to measure changes in a shorter study. Despite the reasonable set of items, it was clear that the survey was not designed to assess a single factor or construct. Although the authors reported that the average survey rating (and several individual items) improved as a result of a cognitive training intervention, more specificity may be warranted to interpret those changes.

Over the last several years, the same nine-item survey has been made available to hundreds of thousands of users of the Lumosity cognitive training program (Lumos Labs, Inc., San Francisco, CA) to inform future development of the program. Within this larger group, a subset of individuals has also provided demographic information (age, educational attainment, gender) and aspects of health history, including whether they have been diagnosed with any of a number of medical conditions. We capitalized on the availability of this massive, pre-existing data set to evaluate the original nine-item survey and to formally define and characterize a new instrument with desirable psychometric properties.

To this aim, we present results from four studies that sequentially support the development and validation of the BAMS-7 [see Boateng et al. (2018)]. In Study 1, we hypothesized that exploratory factor analysis of responses from 75,019 healthy individuals in a large, online cohort would reveal distinct, interpretable subscales within the original nine-item survey from Hardy et al. (2015). We expected each subscale to demonstrate acceptable internal consistency and favorable distributional properties. In Study 2, we examined convergent validity of the resulting Attention and Mood subscales in an independent sample of 150 participants from Amazon Mechanical Turk (MTurk), predicting that the BAMS-7 subscales would show significant correlations with established questionnaires measuring similar constructs. In Study 3, we evaluated known-groups validity by comparing BAMS-7 scores from cohorts self-reporting lifetime diagnoses of ADHD, anxiety, or depression (*N* = 12,976; 20,577; and 24,858, respectively) to the healthy cohort. We hypothesized a double dissociation, with the Attention subscale more sensitive to ADHD, and the Mood subscale more sensitive to anxiety and depression. In Study 4, we conducted a confirmatory factor analysis and reanalyzed data from Hardy et al. (2015) to evaluate whether the BAMS-7 subscales were sensitive to change following a cognitive training intervention. We hypothesized that both subscales would detect intervention-related improvement, with larger effects on attention.

## 2 Materials and methods

### 2.1 Participants

Data from six cohorts in four studies were used in the following analyses. Survey responses from healthy participants who originally registered as members of the Lumosity cognitive training program (“Healthy cohort”) were used to develop the BAMS-7 and its subscales in Study 1. Responses from MTurk participants were used to provide convergent validity with existing questionnaires in Study 2. Responses from participants who registered through the Lumosity program and reported that they have been diagnosed with ADHD (“ADHD cohort”), anxiety disorder (“Anxiety cohort”), or depression disorder (“Depression cohort”) were used to evaluate known-groups validity of the BAMS-7 subscales in Study 3. Responses from participants in the experiment run by Hardy et al. (2015) (“Hardy cohort”) were used to identify sensitivity to intervention effects in Study 4. See Table 2 for demographic characteristics of each cohort in each study.

**Table 2:** Demographics of each cohort in the current studies.

	Cohort					
	Healthy	MTurk	ADHD	Anxiety	Depression	Hardy
**N**	75,019	150	12,976	20,577	24,858	3,489
**Age** (range, mean, SD)	18-89 yrs., 49.46, 16.32	20-63 yrs., 40.16, 11.02	18-88 yrs., 42.04, 15.93	18-89 yrs., 47.38, 15.92	18-89 yrs., 49.97, 15.87	18-80 yrs., 37.73, 14.60
**Gender** (%female, %male, %unknown)	57.82, 38.13, 4.05	49.33, 50.00, 0.67	49.22, 46.81, 3.97	68.48, 27.66, 3.86	67.07, 29.36, 3.56	54.46, 45.54, 0
**Education** (bachelor’s or more, less than bachelor’s, unknown)	59.15, 34.36, 6.50	72.00, 28.00, 0	53.61, 40.90, 5.49	54.77, 39.42, 5.81	54.40, 40.08, 5.52	63.63, 34.77, 1.61
**Study #**	1, 3	2	3	3	3	4

Data from the Healthy, ADHD, Anxiety, and Depression cohorts were collected during normal use of a feature of the Lumosity training program. In the Lumosity Privacy Policy (www.lumosity.com/legal/privacy_policy), all participants agreed to the use and disclosure of non-personal data (e.g., de-identified or aggregate data) for any purpose. Participants were included if they were 18–89 years of age. All participants in these cohorts completed an optional survey of medical conditions. The survey began with the question “Have you ever been diagnosed with any of the following medical conditions?” followed by a list of 34 conditions including ADHD, anxiety disorder, and depression. Participants were asked to check off any of the conditions that applied.

Participant cohorts were defined on the basis of their responses to the medical diagnosis question and the completion of their survey items. The Healthy cohort included 75,019 evaluable participants who reported no diagnoses, after excluding 12,979 (14.7%) due to incomplete responses. The ADHD cohort included 12,976 evaluable participants who reported a lifetime diagnosis of ADHD, after excluding 1,824 (12.3%) due to incomplete responses. The Anxiety cohort included 20,577 participants who reported a lifetime diagnosis of anxiety disorder, after excluding 3,037 (12.9%) due to incomplete responses. The Depression cohort included 24,858 participants who reported a lifetime diagnosis of depression disorder, after excluding 3,740 (13.1%) due to incomplete responses. Comorbid conditions were allowed in the ADHD, Anxiety, and Depression cohorts, such that a participant could be in multiple cohorts (see Supplementary Table 1).

Data from the additional MTurk cohort included 150 participants who were 18 or older from the general population and based in the United States, and who passed a set of prespecified attention checks to ensure data quality (see Supplementary material). The MTurk survey required complete responses, so no participants were excluded for incomplete data.

The Hardy cohort included 3,489 participants who participated in the large, online, cognitive training experiment run by Hardy et al. (2015) and who provided complete responses on the nine-item survey used as a secondary outcome measure. Participants ranged in age from 18 to 80. Individuals completed the survey prior to randomization into a cognitive training intervention group or a crossword puzzle active control group, and completed the same survey following the 10-week intervention. A complete description of the cohort and experiment can be found in Hardy et al. (2015). It should be noted that authors of this prior study were employed by Lumos Labs, Inc., as are the authors of the current paper.

For the Healthy, ADHD, Anxiety, Depression, and Hardy cohorts, all data from participants with complete responses was analyzed, and thus the size of the population was very large. Because these were descriptive studies with pre-existing data sets, powering was not calculated; in addition, the size of each population was much larger than is commonly recommended (see (Kyriazos, 2018; Tabachnick and Fidell, 2013)). For the MTurk cohort, which was also a descriptive study, a power analysis indicated that 134 participants would be sufficient to detect correlations between measures with 95% power, two-tailed at *p* < 0.05, with expected correlation strength *r* = 0.30 (G*Power 3.1) (Faul et al., 2009). To account for potential quality issues with remote research platforms such as MTurk (Goodman and Wright, 2022; Chmielewski and Kucker, 2020; Peer et al., 2022), our prespecified methods allowed for recruitment of up to 200 participants, with the expectation that up to 1/3 of the collected data would need to be discarded due to failed attention checks.

An institutional review board [Western-Copernicus Group Institutional Review Board (WCG IRB); Princeton, New Jersey; affiliated with Western-Copernicus Group Clinical, Inc. (WCG Clinical, Inc.); accredited by the Association for the Accreditation of Human Research Protection Programs (AAHRPP); and registered with the Office for Human Research Protections [OHRP and FDA] as IRB00000533] determined that the studies described here were considered exempt research according to the Code of Federal Regulations 46.104; ethics approval and informed consent for the study were waived/exempted by the WCG IRB.

### 2.2 Survey items

Individuals in all six cohorts in the four studies took the original nine-item survey comprising items about cognitive successes and failures, as well as mood, creativity, and concentration. As described in Hardy et al. (2015) and in the Introduction, the first four survey items were similar to items from the CFQ and intended to assess a participant’s cognitive performance over the past month. Each item asked the participant to report the frequency of a certain cognitive failure or success on a Likert scale. Response options were “Never,” “1–2 times during the month,” “1–2 times per week,” “Several times per week,” “Almost every day.” The additional five items related to a participant’s feelings about their mental and emotional state over the past week. Response options for the second group of questions were “Strongly disagree,” “Disagree,” “Neither agree nor disagree,” “Agree,” “Strongly agree.”

Responses concerning cognitive successes and failures referenced the past month, while those related to current state referenced the past week. The one-month window for behavioral items was selected to balance two priorities: (1) providing adequate opportunity for such experiences to occur, and (2) maintaining sensitivity to cognitive changes, especially in the context of intervention studies. Although the CFQ is traditionally administered with a six-month reference period, Broadbent et al. (1982) used a shorter timeframe in a study involving repeated administrations at six-week intervals. In contrast, items reporting on the participant’s feelings about their emotional or mental state did not assess behavior frequency, allowing for a shorter evaluation window. The use of a one-week period aligns with standard practices in assessments with similar items, which typically use a shorter timeframe.

Participants were able to skip an item by selecting “N/A” in Studies 1, 3, and 4; the MTurk sample in Study 2 did not have this option. Only participants who responded to all items were included; scores are only considered valid or complete if there are responses to all items (i.e., no N/A values).

Scoring involves numerically coding each response option on a scale from 0 to 4, where 0 represents the most negative response and 4 represents the most positive response. Items 1 (losing track of details reading), 2 (misplacing keys), 3 (losing concentration), 7 (anxious), 8 (bad mood), and 9 (sad) are reverse scored with 0 representing the most negative response and 4 representing the most positive response. Thus, with this scale, a higher item score denotes better attention or more positive mood, depending on the focus of the question.

### 2.3 Concordance with existing questionnaires

To establish convergent validity with existing questionnaires in Study 2, data from 150 participants were collected via MTurk and analyzed for the BAMS-7. Standard attention checks were included given expected variability in the quality of MTurk participants (Goodman and Wright, 2022; Chmielewski and Kucker, 2020; Peer et al., 2022), as detailed in the Supplementary material. The additional questionnaires included: the 18-item Adult ADHD Self-Report Scale with a 6-month time interval (ASRS) (Kessler et al., 2005), 12-item Attention-Related Cognitive Errors Scale with an open (unspecified) time interval (ARCES) (Cheyne et al., 2006), 9-item Patient Health Questionnaire with a 2-week time interval (PHQ-9) (Kroenke et al., 2001), 20-item Positive and Negative Affect Schedule with a “few-weeks” time interval (PANAS) (Watson et al., 1988), and 7-item Generalized Anxiety Disorder questionnaire with a 2-week time interval (GAD-7) (Spitzer et al., 2006). Standard scoring was adopted for each questionnaire.

#### 2.3.1 ASRS

The three outcome variables are the sum of 9 items for Part A, the sum of 9 separate items for Part B, and the sum of all 18 items for Parts A + B. Each item is rated on a five-point Likert scale (0 = Never, 1 = Rarely, 2 = Sometimes, 3 = Often, 4 = Very Often). A higher sum (ranging from 0 to 36) for Part A denotes more inattention, a higher sum (ranging from 0–36) for Part B denotes more hyperactivity/impulsivity, and a higher sum (ranging from 0 to 72) for Parts A + B denotes more inattention and hyperactivity/impulsivity (Kessler et al., 2005).

#### 2.3.2 ARCES

The outcome variable is the item mean score across the 12 items. Each item is rated on a five-point Likert scale (1 = Never, 2 = Rarely, 3 = Sometimes, 4 = Often, 5 = Very Often). A higher average (ranging from 1 to 5) denotes more inattention (Cheyne et al., 2006).

#### 2.3.3 PHQ-9

The outcome variable is the sum of the 9 items. Each item is rated on a four-point Likert scale (0 = Not at all, 1 = Several days, 2 = More than half the days, 3 = Nearly every day). A higher sum (ranging from 0 to 27) reflects greater severity of depression, where a score of 1–4 is minimal, 5–9 is mild, 10–14 is moderate, 15–19 is moderately severe, and 20–27 is severe (Kroenke et al., 2001).

#### 2.3.4 PANAS

The two outcome variables are the sum of the 10 positive items and the sum of the 10 negative items, respectively. Each item is rated on a five-point Likert scale (1 = Very slightly or not at all, 2 = A little, 3 = Moderately, 4 = Quite a bit, 5 = Extremely). A higher sum (ranging from 10 to 50) denotes more positive affect or more negative affect, respectively (Watson et al., 1988).

#### 2.3.5 GAD-7

The one outcome variable is the sum of the 7 items. Each item is rated on a four-point Likert scale (0 = Not at all, 1 = Several days, 2 = More than half the days, 3 = Nearly every day). A higher sum (ranging from 0 to 21) reflects greater severity of anxiety, where a score of 0–4 is minimal, 5–9 is mild, 10–14 is moderate, and 15–21 is severe (Spitzer et al., 2006).

The Supplementary material contains additional details regarding the MTurk design, attention checks, and questionnaires.

### 2.4 Statistical approaches

Four core approaches (Beavers et al., 2013; Brysbaert, 2024; Costello and Osborne, 2005; Knekta et al., 2019) were implemented to establish the BAMS-7. First, exploratory factor analysis was run for the Healthy cohort in Study 1 to identify the items for the BAMS-7 and its subscales. To address collinearity and sampling distribution adequacy, correlations among the items were examined, along with the Bartlett sphericity (Bartlett, 1950) and Kaiser-Meyer-Olkin (KMO) (Kaiser, 1974) test statistics for factorability. Then, parallel analysis (Horn, 1965) was used to find the ideal number of latent factors to extract from the initial nine-item survey. This method is considered a superior method to Kaiser’s criterion (i.e., the Kaiser-Guttman rule) and the gold standard in factor analysis research (Fabrigar and Wegener, 2011; Frazier and Youngstrom, 2007; Hoelzle and Meyer, 2013; Velicer and Fava, 1998; Watkins, 2018). A scree plot was also qualitatively assessed to further support the number of factors to retain. Using the number of latent factors determined in this way, exploratory factor analysis was conducted with minimum residual (MINRES) extraction and varimax (orthogonal) rotation, chosen to prioritize stable estimation of latent constructs and good interpretability. Items that loaded onto each factor with a loading above 0.4 were identified. Cronbach’s alpha was utilized to determine the internal consistency of each factor.

Second, after appropriate removal of two of the nine items and an orphaned factor on the basis of poor interpretability and psychometric characteristics (Guvendir and Ozkan, 2022), convergent validity of the resulting 7-item, 2-factor solution was established in Study 2 by examining correlations between the two subscales and existing, validated questionnaires of attention and mood in the MTurk sample. Third, known-groups validity of the BAMS-7 subscales was evaluated by conducting a receiver operating characteristic (ROC) analysis to data from ADHD, Anxiety, and Depression cohorts in Study 3. Fourth, an analysis of sensitivity to intervention effects was conducted with the Hardy cohort in Study 4, as well as a confirmatory factor analysis in this separate, independent sample. Model fit via Comparative Fit Index (CFI) and covariance between factors via Root Mean Square Error of Approximation (RMSEA) were examined for the confirmatory factor analysis (Browne and Cudeck, 1992).

### 2.5 Statistical analysis

Statistical analyses were primarily conducted in Python (version 3.9.7) using Pandas (version 1.3.5) and NumPy (version 1.20.3) and the following freely available libraries. Exploratory and confirmatory factor analysis used the factor_analyzer library (version 0.3.1). Cronbach’s alpha was computed with Pingouin (version 0.5.2), as was the ANCOVA analysis to reanalyze the original (Hardy et al., 2015) intervention results given the newly defined BAMS-7. Distribution skewness and kurtosis were computed with SciPy (version 1.7.3), as were correlations among questionnaires from the MTurk sample. Receiver operating characteristic (ROC) analysis used Scikit-learn (version 1.0.2). Outside of Python, JASP (version 0.17.2.1) was used to calculate CFI and RMSEA for the confirmatory factor analysis.

Unless otherwise stated, 95% confidence intervals and statistical comparisons were computed using standard bootstrap procedures (Wright et al., 2011) with 10,000 iterations.

## 3 Results

### 3.1 Study 1

#### 3.1.1 Analysis of the original nine-item survey

**Figure 1:**
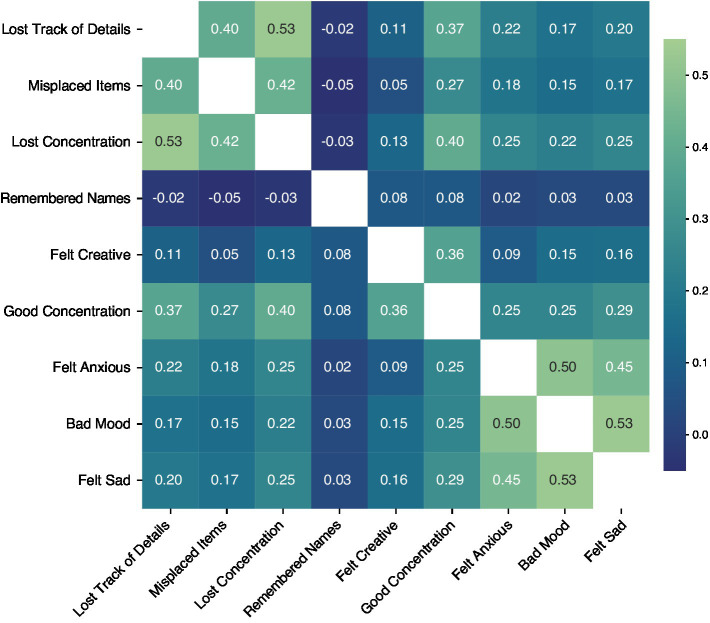
Inter-Item correlation coefficients of the original nine-item survey in Study 1. Warmer colors show stronger correlations between respective items. All correlations are significantly different from 0 with *p*’s < 0.0001.

Survey results from the Healthy cohort (*N* = 75,019) had varying degrees of inter-item (pairwise) correlation, ranging from −0.05 to 0.53, as shown in Figure 1. All correlations were significantly different from 0 (with bootstrapped 95% confidence intervals) at the *p* < 0.0001 level. Item-total correlations ranged from 0.03 (“Remembered Names”) to 0.52 (“Good Concentration”) and were all significantly different from 0 (*p* < 0.0001 for all items). Cronbach’s alpha for the full survey was 0.705 (0.702–0.708). Bartlett’s test of sphericity was significant (*T* = 136408.60, *p* < 0.0001), and the KMO statistic was acceptable at 0.77, above the commonly recommended threshold of 0.70 (Hoelzle and Meyer, 2013; Lloret et al., 2017). These results indicate strong factorability and sampling adequacy in the data set.

**Figure 2:**
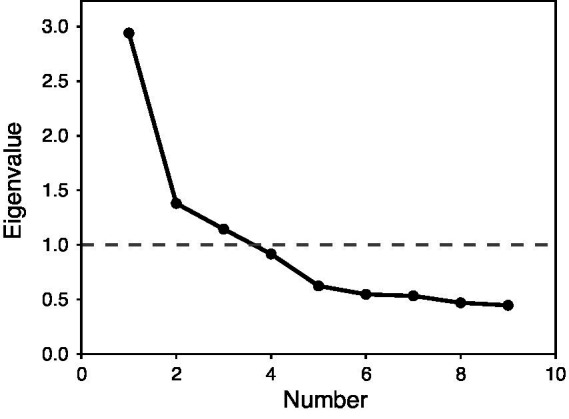
Scree plot for exploratory factory analysis of the original nine-item survey in Study 1. X-axis shows factor number and Y-axis shows eigenvalue. The dotted line with 1.0 is the cutoff for inclusion in the factor solution.

Next, parallel analysis was used to determine the number of latent factors to retain in an exploratory factor analysis, with 41.29% of the variance explained in a resulting 3-factor solution. Additional verification by scree plot is displayed in Figure 2, also indicating 3 factors clearing the eigenvalue threshold of 1.0. The initial results indicate that a 3-factor solution might be appropriate.

Then, the results of the 3-factor solution were computed as shown in Table 3, with factor loadings of 0.4 or greater in bold type. As expected, several of the items related to cognitive successes and failures loaded together, as did several of the items related to mood. The “Remembered Names” item did not load significantly onto any of the three factors, and was dropped. The “Good Concentration” item was the only one to load strongly onto multiple factors: both the first factor, which included other items related to cognitive failures primarily associated with attention functioning, and the third factor, which included the “Felt Creative” item.

**Table 3:** Results of exploratory factor analysis of original nine-item survey in Study 1.

Item	Factor 1	Factor 2	Factor 3
Lost track of details	**0.69**	0.13	0.04
Misplaced items	**0.56**	0.12	−0.04
Lost concentration	**0.72**	0.18	0.07
Remembered names	−0.06	0.02	0.18
Felt creative	0.11	0.09	**0.52**
Good concentration	**0.46**	0.19	**0.56**
Felt anxious	0.19	**0.63**	0.05
In bad mood	0.09	**0.77**	0.12
Felt sad	0.16	**0.65**	0.16

Factor loadings of 0.4 or higher are in bold type.

Cronbach’s alpha was computed to assess the internal consistency of each factor in the 3-factor solution. Factors 1 and 2 both had acceptable Cronbach’s alpha values of 0.728 and 0.745, respectively, with bootstrapped 95% confidence intervals of 0.725–0.731 and 0.742–0.748. Factor 3, however, had a lower Cronbach’s alpha value of 0.529, with a bootstrapped 95% confidence interval of 0.522–0.535.

Although parallel analysis suggested a 3-factor solution, the third factor exhibited poor internal consistency (Cronbach’s alpha = 0.529) and lacked a coherent interpretation, motivating a more parsimonious 2-factor solution. The third factor was eliminated, as was the orphaned item (“Felt Creative”) that no longer loaded onto a factor. This approach is consistent with best practices that recommend considering both statistical and conceptual criteria when determining factor retention (Costello and Osborne, 2005; Hoelzle and Meyer, 2013).

#### 3.1.2 Characterization of the BAMS-7

The resulting seven-item, two-factor scale is the BAMS-7, shown in Table 4. On the basis of the factor analysis and the nature of the items, scores from items loading onto the first factor are averaged to compute an Attention subscale, and scores from items loading onto the second factor are averaged to compute a Mood subscale. Note that the elimination of two of the original items now allowed for a more precise interpretation of the resulting subscales than the original description in Hardy et al. (2015) (i.e., “attention and mood” rather than the broader “cognitive performance and emotional status”).

**Table 4:** Characterization of the BAMS-7 and its subscales from Study 1.

Item	During the past month, how often have you… *Valid response options: “Never,” “1–2 times during the month,” “1–2 times per week,” “Several times per week,” “Almost every day”*	Subscale
1	…lost track of details as you were reading and needed to go back and reread sections?	Attention
2	…misplaced items (e.g., reading glasses, keys) around the house?	Attention
3	…found yourself losing concentration during a conversation?	Attention

	Rate your experience over the last week… *Valid response options: “Strongly disagree,” “Disagree,” “Neither agree nor disagree,” “Agree,” “Strongly agree”*	
4	My ability to concentrate was good.	Attention
5	I felt anxious.	Mood
6	I was in a bad mood.	Mood
7	I felt sad for no obvious reason.	Mood

Note also that the two groups of question types in the BAMS-7 do not correspond directly to the two factors. Instead, the item on “Good Concentration,” despite falling in the second group of questions because it relates to the participant’s current state, loads onto the first factor (factor loading of 0.46) rather than the second (factor loading of 0.19) and therefore contributes to the Attention subscale.

Distributional and psychometric properties of the BAMS-7 subscales are shown in Table 5 for the Healthy cohort. Both of the subscales have modest, but statistically significant, negative skewness and kurtosis.

**Table 5:** Distributional and psychometric properties of the BAMS-7 in Study 1.

Characteristics	Factor 1: Attention subscale	Factor 2: Mood subscale
Mean	2.3856 (2.3795, 2.3919)	2.2359 (2.2288, 2.2428)
SD	0.8598	0.9836
Range	0–4	0–4
Skew	−0.4837 (−0.4955, −0.4721)	−0.1399 (−0.1508, −0.1291)
Kurtosis	−0.3624 (−0.3850, −0.3396)	−0.6942 (−0.7082, −0.6805)

Bootstrapped 95% confidence intervals are shown in parentheses as applicable. Skew and kurtosis are significantly different from 0 with *p*’s < 0.0001.

Both of the subscales are related to the demographic variables of gender and age in some way. With one-way ANOVAs, the Attention subscale significantly varied with gender (*Mean_Male_* = 2.3608, *Mean_Female_* = 2.4011, *Mean_Unknown_* = 2.3981; *F* = 19.31, *p* < 0.0001) while the Mood subscale did not (*Mean_Male_* = 0.9727, *Mean*_*Femal*e_ = 0.9912, *Mean_Unknown_* = 0.9785; *F* = 1.33, *p* = 0.27). With correlation tests, both subscales were positively associated with age within the measured range (18–89 years) (Attention: *r* = 0.1994, *p* < 0.001; Mood: *r* = 0.2285, *p* < 0.001). While an age-related increase on the Attention scale may be surprising given the well-established decline in cognitive performance during aging, this finding is consistent with the characteristics of the CFQ [see Rast et al. (2009) and de Winter et al. (2015); for similar results with additional questionnaires, see Cyr and Anderson (2019) and Tassoni et al. (2022)]. It is also consistent with the hypothesis that self-reported cognitive failures and successes may reflect something distinct from what is measured via objective cognitive tests (Eisenberg et al., 2019; Yapici-Eser et al., 2021).

#### 3.1.3 Norms for the BAMS-7

**Figure 3:**
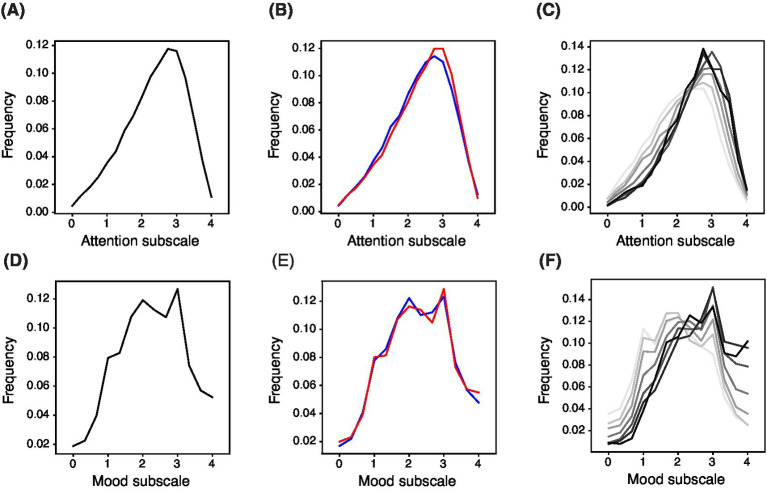
Normed Distribution of BAMS-7 Attention and Mood Subscales across the Whole Population, by Gender, and by Age in Decade. Panels **(A)** and **(D)** refer to the whole normed distribution of ages and genders for Attention and Mood subscales, respectively. Panels **(B)** and **(E)** refer to distribution by gender (red = female, blue = male) across ages. Panels **(C)** and **(F)** refer to distribution by age in decade (darker color is older) across genders.

A strength of the BAMS-7 is the scale of its normative data set. Normative distributions are shown across the whole population of 75,019 healthy participants for the Attention subscale (Figure 3A) and Mood subscale (Figure 3D), by gender (Figures 3B,E), and by age in decade (Figures 3C,F).

Norm tables providing percentiles and standardized T-scores across the whole population, by gender, and by age in decade are also provided in look-up format for the Attention subscale (Tables 6A,B) and Mood subscale (Tables 6C,D). T-scores provide conversions to a normal distribution with mean 50, standard deviation 10, and bounds at 20 and 80.

**Table 6:** Percentile and T-score norm tables for the attention and mood subscales across the whole population, by gender, and by age in decade.

(A) Attention subscale percentiles																		
Population	*N*	0	0.25	0.5	0.75	1	1.25	1.5	1.75	2	2.25	2.5	2.75	3	3.25	3.5	3.75	4
Healthy cohort (all genders, ages 18–89)	75,019	0	2	3	6	10	14	20	27	35	45	55	67	79	89	95	99	100
Ages 18–29, gender f	5,059	1	3	6	11	16	22	29	38	47	58	68	79	88	94	98	100	100
Ages 18–29, gender m	7,171	1	2	6	9	15	21	30	39	49	59	69	79	88	94	97	99	100
Ages 30–39, gender f	4,456	1	3	5	9	14	19	26	35	45	55	65	75	86	93	97	100	100
Ages 30–39, gender m	4,712	1	2	4	7	11	17	24	32	41	51	62	73	84	91	97	99	100
Ages 40–49, gender f	6,650	1	2	4	8	12	17	23	30	39	49	59	70	82	91	97	99	100
Ages 40–49, gender m	4,419	0	1	3	5	9	13	20	26	35	45	56	68	79	89	95	99	100
Ages 50–59, gender f	11,662	0	2	3	5	9	13	18	25	33	42	53	65	78	88	95	99	100
Ages 50–59, gender m	5,215	0	1	3	5	8	11	16	22	30	40	51	63	75	86	94	99	100
Ages 60–69, gender f	10,931	0	1	2	4	6	9	14	19	26	35	46	59	72	84	94	98	100
Ages 60–69, gender m	4,702	0	1	2	4	6	9	13	18	26	36	47	59	72	84	93	98	100
Ages 70–79, gender f	4,034	0	1	1	3	5	7	12	18	25	34	45	59	71	84	94	99	100
Ages 70–79, gender m	2020	0	1	2	4	6	9	14	20	28	37	49	61	72	83	93	98	100
Ages 80–89, gender f	584	0	1	2	4	7	9	12	19	28	37	47	60	74	84	93	99	100
Ages 80–89, gender m	368	1	1	2	4	4	8	14	20	27	39	52	67	78	87	95	98	100

(B) Attention subscale T-scores																		
Population	N	0	0.25	0.5	0.75	1	1.25	1.5	1.75	2	2.25	2.5	2.75	3	3.25	3.5	3.75	4
Healthy cohort (all genders, ages 18–89)	75,019	20	24	29	32	34	37	39	42	44	46	49	51	54	58	62	67	73
Ages 18–29, gender f	5,059	20	26	32	35	37	40	42	44	47	49	52	55	58	62	66	70	78
Ages 18–29, gender m	7,171	20	25	30	34	37	40	42	45	47	50	52	55	58	62	65	69	75
Ages 30–39, gender f	4,456	20	27	31	34	36	39	41	44	46	49	51	54	57	61	65	70	76
Ages 30–39, gender m	4,712	20	25	29	32	35	38	40	43	45	48	50	53	56	60	64	68	74
Ages 40–49, gender f	6,650	20	25	30	33	36	38	40	43	45	47	50	52	55	59	63	68	74
Ages 40–49, gender m	4,419	20	22	27	31	34	37	39	41	44	46	49	51	55	58	62	67	72
Ages 50–59, gender f	11,662	20	24	28	31	34	36	39	41	43	46	48	51	54	58	62	66	73
Ages 50–59, gender m	5,215	20	23	28	31	33	36	38	40	42	45	47	50	53	57	61	66	72
Ages 60–69, gender f	10,931	20	21	26	29	32	34	37	39	41	44	46	49	52	56	60	65	72
Ages 60–69, gender m	4,702	20	21	27	30	32	34	37	39	41	44	46	49	52	56	60	64	71
Ages 70–79, gender f	4,034	20	19	25	28	31	33	35	38	41	43	46	49	52	56	60	65	72
Ages 70–79, gender m	2020	20	20	26	29	32	35	37	39	42	44	47	50	53	56	60	65	71
Ages 80–89, gender f	584	20	20	26	30	33	35	37	38	41	44	47	49	53	56	60	65	72
Ages 80–89, gender m	368	20	25	26	30	32	33	36	39	42	44	47	50	54	58	61	67	70

(C) Mood subscale percentiles														
Population	*N*	0	0.33	0.67	1	1.33	1.67	2	2.33	2.67	3	3.33	3.67	4
Healthy cohort (all genders, ages 18–89)	75,019	2	4	8	16	24	35	47	58	69	82	89	95	100
Ages 18–29, gender f	5,059	5	10	17	31	42	53	65	75	84	92	95	98	100
Ages 18–29, gender m	7,171	3	6	12	22	31	43	56	67	77	87	93	97	100
Ages 30–39, gender f	4,456	3	7	13	25	35	48	60	70	79	89	95	98	100
Ages 30–39, gender m	4,712	2	5	10	19	29	41	54	65	75	87	93	97	100
Ages 40–49, gender f	6,650	3	5	10	20	29	41	53	65	74	86	92	97	100
Ages 40–49, gender m	4,419	2	4	8	16	25	37	50	61	72	85	92	96	100
Ages 50–59, gender f	11,662	2	3	7	14	21	32	44	56	67	81	89	95	100
Ages 50–59, gender m	5,215	1	3	6	13	22	32	44	56	67	80	89	95	100
Ages 60–69, gender f	10,931	1	2	5	10	17	26	37	49	60	75	84	92	100
Ages 60–69, gender m	4,702	1	2	4	10	17	26	37	47	59	75	84	93	100
Ages 70–79, gender f	4,034	1	2	4	8	14	23	33	44	56	71	81	90	100
Ages 70–79, gender m	2020	1	2	4	8	14	21	32	42	55	69	80	91	100
Ages 80–89, gender f	584	1	2	4	7	14	24	34	47	60	73	81	89	100
Ages 80–89, gender m	368	0	1	2	7	14	23	35	46	57	71	82	92	100

(D) Mood subscale T-scores														
Population	*N*	0	0.33	0.67	1	1.33	1.67	2	2.33	2.67	3	3.33	3.67	4
Healthy cohort (all genders, ages 18–89)	75,019	20	29	33	36	40	43	46	49	52	55	59	62	66
Ages 18–29, gender f	5,059	20	33	37	41	45	48	51	54	57	60	64	67	71
Ages 18–29, gender m	7,171	20	31	35	38	42	45	48	51	54	58	61	65	69
Ages 30–39, gender f	4,456	20	32	35	39	43	46	50	53	55	58	63	66	70
Ages 30–39, gender m	4,712	20	30	33	37	41	44	48	51	54	57	61	65	69
Ages 40–49, gender f	6,650	20	30	34	37	41	44	48	51	54	57	61	64	68
Ages 40–49, gender m	4,419	20	29	32	36	40	43	47	50	53	56	60	64	68
Ages 50–59, gender f	11,662	20	28	32	35	39	42	45	49	52	55	59	62	66
Ages 50–59, gender m	5,215	20	27	31	35	39	42	45	49	51	55	58	62	66
Ages 60–69, gender f	10,931	20	27	30	33	37	40	44	47	50	53	57	60	64
Ages 60–69, gender m	4,702	20	26	29	33	37	40	44	47	49	52	57	60	64
Ages 70–79, gender f	4,034	20	26	29	32	36	39	43	46	48	51	56	59	63
Ages 70–79, gender m	2020	20	25	29	32	36	39	42	45	48	51	55	58	63
Ages 80–89, gender f	584	20	27	30	32	35	39	43	46	49	52	56	59	62
Ages 80–89, gender m	368	20	22	27	29	35	39	43	46	49	52	55	59	64

For each table, each row is a different slice of the population (or the whole population with all genders, at the top of each table). Tables **(A,C)** show the percentile, i.e., what percent of the population is less than or equal to the value shown, associated with each possible value of the respective subscale. Tables **(B,D)** show the corresponding standardized T-scores, which are defined to have a mean of 50, standard deviation of 10, and bounds at 20 and 80.

### 3.2 Study 2

#### 3.2.1 Concordance with existing questionnaires

To establish convergent validity, a series of correlations were computed relating the BAMS-7 Attention and Mood subscales to five known instruments of attention and mood over various timescales from the independent MTurk cohort. Each of these previously validated instruments had good or excellent internal consistency within this cohort (Supplementary Table 2). Because these other instruments were selected to capture conceptually overlapping constructs, moderate to high correlations with the BAMS-7 subscales were expected and considered evidence of convergent validity.

Table 7A shows *r*-values from pairwise correlations between the BAMS-7 Attention subscale and the attention instruments, and Table 7B shows *r*-values for the BAMS-7 Mood subscale with the mood instruments. All *p*-values for the correlations were < 0.001, meaning that the BAMS-7 Attention and Mood subscales showed significant relationships, respectively, with each existing questionnaire of attention and mood. The Attention subscale showed stronger relationships numerically with the attention instruments – ASRS and ARCES – while the Mood subscale showed stronger relationships with the mood instruments – GAD, PHQ, and PANAS. Note that many of the correlations are negative because a higher score on the BAMS-7 indicates better attention or mood while a higher score on each of the known instruments (excluding PANAS positive affect) indicates higher inattention or lower mood.

**Table 7:** Convergent Validity of BAMS-7 **(A)** Attention subscale with existing attention questionnaires and **(B)** Mood subscale with existing mood questionnaires in Study 2.

(A)	
Questionnaire	BAMS-7 Attention subscale
ASRS Part A (Inattention)	−0.750
ASRS Part B (Hyperactivity/Impulsivity)	−0.609
ASRS Total (A + B)	−0.720
ARCES	−0.788

(B)	
Questionnaire	BAMS-7 Mood subscale
PHQ	−0.770
PANAS Pos Aff	0.320
PANAS Neg Aff	−0.780
GAD	−0.765

For each table, correlation coefficients are shown and *p*’s < 0.001.

This pattern of results indicates that the BAMS-7 shows concordance with existing questionnaires. This can be seen in Supplementary Table 3, which shows the entire set of correlations between both BAMS-7 subscales and all five existing attention and mood instruments [for similar results, see Carriere et al. (2008), Franklin et al. (2017), Jonkman et al. (2017), and Schubert et al. (2024)]. The Supplementary material also contains an additional Supplementary Table 4 demonstrating strong item-level correlations between each of the BAMS-7 questions and those from the existing questionnaires with similar descriptions, demonstrating additional concordance at the item level.

### 3.3 Study 3

#### 3.3.1 Discriminatory power of the subscales in ADHD, anxiety, and depression

**Figure 4:**
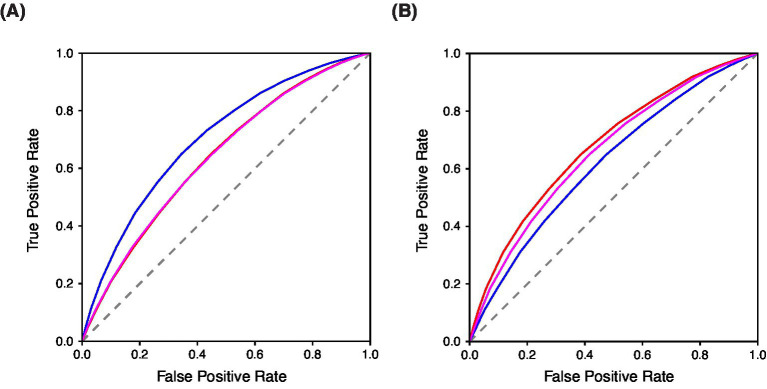
Known-groups validity evaluated with ROC Curves of BAMS-7 (A) Attention Subscale and (B) Mood Subscale in ADHD, anxiety, and depression cohorts in Study 3. In each figure, blue = ADHD cohort, red = Anxiety cohort, and magenta = Depression cohort.

To evaluate the convergent and divergent validity of the BAMS-7, Attention and Mood subscale scores from the ADHD, Anxiety, and Depression cohorts were each compared to those from the Healthy cohort. A series of ROC analyses were performed to assess known-groups validity: (1) Attention subscale scores for ADHD vs. Healthy, (2) Attention subscale scores for Anxiety vs. Healthy, (3) Attention subscale scores for Depression vs. Healthy, (4) Mood subscale scores for ADHD vs. Healthy, (5) Mood subscale scores for Anxiety vs. Healthy, and (6) Mood subscale scores for Depression vs. Healthy. The resulting ROC curves are shown in Figure 4A for the Attention subscale and Figure 4B for the Mood subscale, and the corresponding areas under the curves (AUCs) are shown in Table 8.

**Table 8:** Known-groups validity with AUC Properties of BAMS-7 attention and mood subscales in ADHD, anxiety, and depression cohorts in Study 3.

AUC	Attention subscale	Mood subscale
ADHD vs. Healthy	0.7040 (0.6993, 0.7088)	0.6196 (0.6144, 0.6247)
Anxiety vs. Healthy	0.6392 (0.6351, 0.6435)	0.6795 (0.6755, 0.6835)
Depression vs. Healthy	0.6391 (0.6217, 0.6408)	0.6587 (0.6549, 0.6626)

Bootstrapped 95% confidence intervals are shown in parentheses.

Differences within each of the three psychiatric conditions vs. healthy controls were assessed by subscale to examine discriminatory ability. Within ADHD vs. Healthy, the Attention subscale had a significantly higher AUC than the Mood subscale (0.7040 for the Attention subscale and 0.6196 for the Mood subscale, *p* < 0.0001), which provides further evidence of the factor structure of the BAMS-7, given that ADHD is primarily a disorder of attention (Kessler et al., 2005). For both the Anxiety vs. Healthy and Depression vs. Healthy comparisons, it was instead the Mood subscale that was significantly better at discriminating between populations compared to the Attention subscale (Anxiety vs. Healthy: 0.6795 for the Mood subscale and 0.6392 for the Attention subscale, *p* < 0.0001; Depression vs. Healthy: 0.6587 for the Mood subscale and 0.6391 for the Attention subscale, *p* < 0.0001). This profile provides additional validation of the meaning of the subscales because mood is a hallmark of anxiety and depression (Kroenke et al., 2001; Spitzer et al., 2006).

The ability of each of the BAMS-7 subscales to discriminate between the three psychiatric populations was assessed. Indeed, for the Attention subscale, the AUC was significantly greater for the ADHD vs. Healthy analysis relative to each of the Anxiety and Depression vs. Healthy contrasts (0.7040 compared to 0.6392 and 0.6391, respectively, *p* < 0.0001 for each comparison). There was no significant difference between the AUC of the Anxiety vs. Healthy and Depression vs. Healthy analysis for the Attention subscale. Conversely, the Mood subscale had the poorest discrimination (i.e., lowest AUC) for ADHD vs. Healthy. Instead the Mood subscale had the highest AUC for the Anxiety vs. Healthy analysis (0.6795), followed by Depression vs. Healthy (0.6587), followed by ADHD vs. Healthy (0.6196), *p* < 0.0001 for each comparison.

### 3.4 Study 4

#### 3.4.1 Sensitivity to a cognitive intervention

To confirm the factorization of the BAMS-7 and to test whether the BAMS-7 might have utility as an outcome measure in studies, we re-analyzed the data from the Hardy et al. (2015) experiment using the new characterization of the BAMS-7 and its Attention and Mood subscales, excluding any participants with incomplete data. Our confirmatory factor analysis indicated adequate model fit of the BAMS-7 (CFI = 0.98 and RMSEA = 0.05), with CFI greater than 0.90 and RMSEA less than 0.08 (Browne and Cudeck, 1992). The loadings of the seven items were also adequate and above 0.40 for the Attention subscale (item 1/rereading = 0.72, item 2/misplacing items = 0.60, item 3/losing concentration = 0.87, and item 4/good concentration = 0.48) and for the Mood subscale (item 5/anxious = 0.73, item 6/bad mood = 0.83, and item 7/sad = 0.84).

After confirming that the BAMS-7 is not sample-dependent via the confirmatory factor analysis, we followed the original analysis of Hardy et al. (2015) by implementing a statistical analysis with an ANCOVA. Change in BAMS-7 subscale score (follow-up - baseline) was the dependent variable, intervention group (cognitive training or an active control) was the grouping variable, and baseline score was a covariate. Age was also included as a covariate to examine differential effects of intervention across the lifespan (for covariate results, see the Supplementary material).

Table 9 shows the results of the analysis of the Hardy cohort on the BAMS-7 Attention and Mood subscales. Consistent with the original analysis, there was a group (intervention) effect on the change in both the Attention and Mood subscales with the cognitive training group improving more than the active control one [Attention: *F*(1,3,485) = 53.73, *p* < 0.0001; Mood: *F*(1,3,485) = 17.57, *p* < 0.001]. However, as might be expected for a cognitive intervention, the effect size (Cohen’s *d* of ANCOVA-adjusted change scores) was greater for the Attention subscale (0.247) than for the Mood subscale (0.148). These results demonstrate that the subscales of the BAMS-7 are sensitive to a cognitive intervention, and therefore may have utility as outcome measures in studies.

**Table 9:** Sensitivity of BAMS-7 subscales to intervention effects using data from Hardy et al. (2015).

Intervention effect	Active control	Cognitive training
Attention subscale Change mean (SD)	0.4741 (0.7186)	0.6022 (0.7550)
Mood subscale Change mean (SD)	0.3250 (0.9429)	0.4390 (0.9204)

## 4 Discussion

We describe a brief, seven-item scale of real-world attention and mood established from a very large, real-world data set: the BAMS-7. The scale is specifically designed and validated for at-home, self-administration, emphasizing brevity and accessibility—key priorities in the current research landscape—and shows potential for assessing multiple constructs effectively (Aiyegbusi et al., 2024; Aiyegbusi et al., 2022; Gottfried, 2024; Kelfve et al., 2020; Lantos et al., 2023; Zager Kocjan et al., 2023; Wilson et al., 2024).

Four studies establish the validity and reliability of the BAMS-7. The scale was developed in Study 1 using data from 75,019 healthy individuals who participated in the Lumosity cognitive training program; Study 1 was also used to characterize the inter-item correlation coefficients of the original nine items of the initial survey, and to determine Cronbach’s alpha and establish distributional and psychometric properties of the BAMS-7. Concordance with existing scales for attention and mood was established in Study 2 using data from an MTurk sample. Study 3 established known-groups validity in cohorts reporting lifetime diagnoses of conditions that might be expected to have specific impairments on one or the other BAMS-7 subscale (ADHD on the Attention subscale and anxiety or depression on the Mood subscale). Study 4 re-examined data from a large-scale cognitive training study published by Hardy et al. (2015) with a confirmatory factor analysis to determine whether the BAMS-7 subscales may be sensitive to cognitive interventions.

Factor analysis indicates two latent factors in the seven-item scale. Items assessing the first factor include adaptations of three items from the CFQ that focus on real-world attention function, and one item that queries the extent to which the responder agrees with the statement “I had good concentration” over the past week. The second factor includes items related to mood and anxiety. The resulting subscales – Attention and Mood, respectively – have acceptable internal consistency and descriptive statistics that may make them useful in research.

A strength of the BAMS-7 is the size and diversity of its normative data set. Age norms in the range 18–89 are provided, along with normed distribution and look-up tables across the whole population, by gender, and by age in decade for each subscale. These norms have potential to assist in comparisons from study to study and in standardized effect sizes, along with the identification of outliers (Brysbaert, 2024). It should be noted, though, that participants in several of the analysis cohorts were users of the Lumosity cognitive training program and may have had certain psychographic similarities (Hardcastle and Hagger, 2016) despite the broad demographic representation. The MTurk cohort (Study 3) did not have this limitation, and future studies in other, unrelated populations are encouraged.

Both the Attention and Mood subscales are positively correlated with age, which may appear paradoxical given the extensive literature on age-related cognitive decline. However, this relationship with age is consistent with the CFQ [e.g., Rast et al. (2009) and de Winter et al. (2015)], suggesting a general divergence between objective and subjective measures of cognitive performance. It should be noted that the correlation with age is observed on a cross-sectional basis, so an alternative hypothesis is that there are generational differences in the perception of cognitive functioning. Future research should determine how subjective cognitive measures like the BAMS-7 change in longitudinal studies.

There are at least a few questions that stem from the current work on the BAMS-7. First, is it really necessary to have another psychometrically validated scale of this kind? The existing scales used in Study 2, for example, are relatively brief, validated, and commonly used in research or clinical practice. Despite the existence of other viable options, we think there remains an opportunity for instruments that are defined and validated entirely for self-administered, online use. Additionally, the scale of the available normative data set may provide advantages over existing scales for certain uses or populations. Second, is it okay that time intervals are different between items assessing the frequency of attentional successes and failures (evaluated over the past month), and those related to one’s current feelings or emotional state (evaluated over the last week)? We think that it makes sense to consider different time intervals for these two types of items for two reasons: first, fluctuations that are significant in mood and attention may not operate on the same time scales (Irrmischer et al., 2018; Esterman and Rothlein, 2019; McConville and Cooper, 1997; Rosenberg et al., 2020; Zanesco et al., 2022). Second, items based on the frequencies of certain real-world behaviors – such as cognitive successes or failures – should use time intervals long enough to provide sufficient opportunities for measurement, whereas items based on a belief or emotion do not have the same requirement. The BAMS-7 includes both types of items.

A limitation of the work is that known-groups validation of the BAMS-7 may be constrained by the fact that the ADHD, Anxiety, and Depression cohorts were defined by self-reports of a lifetime clinical diagnosis. It is worth noting, however, that a similar survey-based determination of ADHD has been used by the CDC to assess the prevalence of adult ADHD (Staley et al., 2024), and that prior studies have concluded validity of self-reported medical diagnosis in other psychiatric conditions (Santos et al., 2021; Woolway et al., 2024). Nevertheless, methodology for reporting diagnosis may relate to the AUC values that were obtained in Study 3: while there is no consensus threshold for adequacy of AUC values, some research has suggested that only values above 0.70 represent adequate discrimination [e.g., Hosmer and Lemeshow (2000)]. Most of the AUC values were modest and hovered under this cut-off. We think it is possible that the classification performance reported here is underestimated given constraints of the sample. Date of diagnosis was not reported, nor was current symptom or treatment status. If some individuals within the ADHD, Anxiety, and Depression cohorts were receiving treatment or otherwise without symptoms when taking the BAMS-7, it may be surprising that the BAMS-7 subscales could successfully discriminate at all between the three cohorts.

Interestingly, despite the modest overall AUC values, the BAMS-7 Attention subscale exhibited slightly elevated and statistically significant AUCs for individuals with anxiety and depression (relative to healthy participants), and the BAMS-7 Mood subscale showed a similar elevation for individuals with ADHD. These patterns may reflect the real-world occurrence of comorbidities within these clinical cohorts: for instance, individuals with both ADHD and depression may have been included in both diagnostic groups (see Supplementary Table 1). Alternatively, the elevated AUCs may highlight the known interdependence between attention and mood-related processes in psychiatric conditions (D’Agati et al., 2019; Retz et al., 2012; Schnyer et al., 2015; Gkintoni and Ortiz, 2023; Keller et al., 2019; Williams, 2016). Future studies employing more precisely defined clinical samples, including data on current symptom severity and treatment status, will help clarify these effects.

Another limitation of the work is that the nine-item survey from which the BAMS-7 was derived was not systematically developed from an initial item pool using a deductive or inductive approach (see (Boateng et al., 2018)). Some of the items stem from the CFQ, but a formal item development process was not adopted for the nine-item survey itself. Instead, the development and characterization of the BAMS-7 was motivated by the pre-existence of a massive data set. Aside from the constraints of the initial pool of items, a standard and rigorous process for scale development was followed.

Overall, the pattern of results indicates that a self-administered, brief, accessible, online instrument measuring multiple constructs may be useful in the current research landscape (Bowling, 2005; Rolstad et al., 2011). The BAMS-7 and its large normative data set show promise for improving measurement and understanding of cognition and mood in the social and clinical sciences.

## Data Availability

The original contributions presented in the study are included in the article/Supplementary material, further inquiries can be directed to the corresponding author/s.
