# Supplementary material for: Reliability and validity of the Brief Attention and Mood Scale of 7 Items: a self-administered, online assessment
Source: Front Psychol. 2025 Oct 1;16:1579235. doi: 10.3389/fpsyg.2025.1579235 (PMC12520896; doi:10.3389/fpsyg.2025.1579235)
Supplement: Supplementary file 1 [file Supplementary_file_1.docx]

Supplementary Material

# 1 Study 3

# Supplementary Table 1. *Number of Participants by Cohort Condition in Study 3.*

| **Condition** | **Number of Participants** |
| --- | --- |
| ADHD only | 7,057 |
| Anxiety only | 9,268 |
| Depression only | 13,325 |
| ADHD + Anxiety only | 1,769 |
| ADHD + Depression only | 1,993 |
| Anxiety + Depression only | 7,383 |
| ADHD + Anxiety + Depression | 2,157 |

**2 Study 2**

***2.1 MTurk Design for Concordance***

We intended to recruit up to 200 individuals ages 18 and older who resided in the United States for a 7 min online Amazon MTurk HIT (human intelligence task) of “Cognition and Emotion” that paid $1.00. Various attention checks in the task reduced the sample from 200 to 150 participants for analysis (i.e., 1/4 of participants were discarded rather than our larger projection of 1/3). The attention checks involved identifying response inconsistencies, random clicking, too little or too long spent on HIT, and bots. One of the attention checks involved having participants re-rate five items from across the different questionnaires; participants were excluded if their average response mismatch was 1-point or more. After demographics, questionnaires included the ASRS, ARCES, Hardy survey (with emphasis on BAMS-7 items), PHQ-9, PANAS, and GAD-7.

***2.2 Questionnaire Descriptions for Concordance***

**2.2.1 Adult ADHD Self-Report Scale** (ASRS) [53]. The ASRS symptom checklist from the World Health Organization measures for probable ADHD in adults as well as ADHD symptoms. The checklist asks respondents to indicate how they have felt and conducted themselves over the past 6 months in terms of frequency of inattention and hyperactivity/impulsivity symptoms.

**2.2.2 Attention-Related Cognitive Errors Scale** (ARCES) [54]. The ARCES measures the frequency of cognitive errors in everyday situations that are attributed to attention lapsing. It is well-validated and reliable for remote assessment in populations across the lifespan [42, 54, 94]. Score on the ARCES is related to self-reported cognitive and clinical outcomes from independent questionnaires, including memory failures, boredom, fidgeting, mind wandering, daydreaming, media multitasking, lack of attentional control, and symptoms of depression and ADHD [79, 80, 95]. Score on the ARCES is related to task-based continuous performance test (CPT) commission errors [96], and psychometric work has shown that it is also separable from that of the CFQ [97].

**2.2.3 Patient Health Questionnaire** (PHQ-9) [55]. The PHQ-9 measures for probable major depressive episodes as well as depressive symptom severity. Each item represents one of the diagnostic criteria for major depressive episodes. The PHQ-9 asks participants to report the presence of each symptom within the last 2 weeks.

**2.2.4 Positive and Negative Affect Schedule** (PANAS) [56]. The PANAS measures positive and negative affect along various emotion and mood dimensions over various time-sensitive intervals, including over the past few weeks. The positive affect score and negative affect score are separable. It is well-validated and reliable for clinical and social sciences.

**2.2.5 Generalized Anxiety Disorder** (GAD-7) [57]. The GAD-7 measures for probable generalized anxiety disorder as well as anxiety symptom severity. The GAD-7 asks participants to report the presence of each symptom within the last 2 weeks.

**2.3 MTurk Results for Concordance**

# Supplementary Table 2. *Internal Consistency (Cronbach’s alpha) of the Instruments Used for Concordance Analysis in the MTurk Cohort.*

| **Measure** | **Internal consistency (Cronbach’s alpha)** |
| --- | --- |
| ASRS Part A | 0.885 |
| ASRS Part B | 0.852 |
| ASRS Total (A+B) | 0.927 |
| ARCES | 0.953 |
| PHQ-9 | 0.858 |
| PANAS Pos Aff | 0.943 |
| PANAS Neg Aff | 0.897 |
| GAD-7 | 0.903 |

# Supplementary Table 3. *Questionnaire Correlation Matrix of the BAMS-7 Attention and Mood Subscales with the Five Existing Instruments (ASRS, ARCES, PHQ-9, PANAS, and GAD-7) in Study 2. Note: *p<*.05*, **p<*.01*, ***p<*.001*, ^△^p<*.10*.*

|  | **BAMS-7 Attention subscale** | **BAMS-7 Mood subscale** | **ASRS Part A** | **ASRS**  **Part B** | **ASRS**  **Total (A+B)** | **ARCES** | **PHQ-9** | **PANAS**  **Pos Aff** | **PANAS Neg Aff** | **GAD-7** |
| --- | --- | --- | --- | --- | --- | --- | --- | --- | --- | --- |
| BAMS-7 Attention subscale | - |  |  |  |  |  |  |  |  |  |
| BAMS-7 Mood subscale | 0.685*** | - |  |  |  |  |  |  |  |  |
| ASRS Part A | -0.750*** | -0.659*** | - |  |  |  |  |  |  |  |
| ASRS Part B | -0.609*** | -0.570*** | 0.777*** | - |  |  |  |  |  |  |
| ASRS Total (A+B) | -0.720*** | -0.652*** | 0.942*** | 0.943*** | - |  |  |  |  |  |
| ARCES | -0.788*** | -0.644*** | 0.834*** | 0.715*** | 0.821*** | - |  |  |  |  |
| PHQ-9 | -0.749*** | -0.770*** | 0.708*** | 0.684*** | 0.738*** | 0.718*** | - |  |  |  |
| PANAS Pos Aff | 0.265** | 0.320*** | -0.118 | 0.144^△^ | 0.014 | -0.069 | -0.178* | - |  |  |
| PANAS Neg Aff | -0.685*** | -0.780*** | 0.652*** | 0.662*** | 0.697*** | 0.623*** | 0.847*** | -0.190* | - |  |
| GAD-7 | -0.755*** | -0.765*** | 0.702*** | 0.674*** | 0.730*** | 0.710*** | 0.916*** | -0.158^△^ | 0.833*** | - |

**Supplementary Table 4.** *Item-Level Concordance Analysis with Correlation Matrix of Each BAMS-7 Item and Related Items from the Five Existing Questionnaires (ASRS, ARCES, PHQ-9, PANAS, and GAD-7) in Study 2. Note: All p’s<*.0001*.*

| **BAMS-7** | **ASRS** | **ARCES** | **PANAS** | **PHQ-9** | **GAD-7** |
| --- | --- | --- | --- | --- | --- |
|  |  |  |  |  |  |
| ***During the last month, how often have you…*** | **Over the last 6 months…** | **How frequently have these sorts of things happened to you…** | **Over the past few weeks…** | **Over the last few weeks…** | **Over the last few weeks…** |
| *Lost track of details as you were reading and needed to go back and reread sections?* | - | When reading I find that I have read several paragraphs without being able to recall what I read.  *r* = -0.623 | - | - | - |
|  |  |  |  |  |  |
| *Misplaced items (e.g., reading glasses, keys) around the house?* | How often do you misplace or have difficulty finding things at home or at work?  *r* =  -0.575 | I have absent-mindedly misplaced frequently used objects, such as keys, pens, glasses, etc.  *r* = -0.570 | - | - | - |
|  |  |  |  |  |  |
| *Found yourself losing concentration during a conversation?* | How often do you have difficulty concentrating on what people say to you, even when they are speaking to you directly?  *r* = -0.558 | I have lost track of a conversation because I zoned out when someone else was talking.  *r* = -0.676 | - | - | - |
|  |  |  |  |  |  |
| ***Rate your experience over the last week…*** |  |  |  |  |  |
| *My ability to concentrate was good.* | - | - | Attentive  *r* = 0.598  Alert  *r* = 0.454 | Trouble concentrating on things, such as reading the newspaper or watching television.  *r* = -0.403 | - |
|  |  |  |  |  |  |
| *I felt anxious.* | - | - | Nervous  *r* = 0.711 | - | Feeling nervous, anxious, or on edge.  *r* = -0.642 |
|  |  |  |  |  |  |
| *I was in a bad mood.* | - | - | Irritable  *r* = -0.578 | Feeling bad about yourself - or that you are a failure or have let yourself or your family down.  *r* = -0.470 | - |
|  |  |  |  |  |  |
| *I felt sad for no obvious reason.* | - | - | Upset  *r* = -0.627 | Feeling down, depressed, or hopeless.  *r* = -0.665 | - |

**3 Study 4**

***3.1 ANCOVA Results with Covariates of Baseline Score and Age***

In line with the original ANCOVA analysis [45], the covariate of baseline BAMS-7 on intervention effects was significant. Participants who had lower pre-intervention Attention and Mood scores exhibited greater post-intervention improvements on the Attention subscale in both Lumosity and Crosswords (*F*(1,3485)=1277.94, *p*<.001) and Mood subscale (*F*(1,3485)=1474.06, *p*<.001). There was also a significant effect of the covariate of age on the Mood subscale (*F*(1,3485)=26.57, *p*<.001) but not the Attention subscale *F*(1,3485)=0.27, *p*=.606), in Lumosity and Crosswords. Participants showed greater improvements on the Mood subscale across interventions with increasing age.

**4 Additional References in Supplementary Material**

94. Carriere JS, Seli P, Smilek D. Wandering in both mind and body: individual differences in mind wandering and inattention predict fidgeting. Can J Exp Psychol. 2013;67(1):19–31.

95. Ralph BC, Thomson DR, Cheyne JA, Smilek D. Media multitasking and failures of attention in everyday life. Psychol Res. 2014;78(5):661–9.

96. Rosenberg M, Noonan S, DeGutis J, Esterman M. Sustaining visual attention in the face of distraction: a novel gradual-onset continuous performance task. Atten Percept Psychophys. 2013;75(3):426–39.

97. Smilek D, Carriere JS, Cheyne JA. Failures of sustained attention in life, lab, and brain: ecological validity of the SART. Neuropsychologia. 2010;48(9):2564–70.
